# Prenatal hypoxia increases susceptibility to kidney injury

**DOI:** 10.1371/journal.pone.0229618

**Published:** 2020-02-21

**Authors:** Kasey R. Cargill, Takuto Chiba, Anjana Murali, Elina Mukherjee, Elizabeth Crinzi, Sunder Sims-Lucas

**Affiliations:** Department of Pediatrics, Division of Nephrology, UPMC Children’s Hospital of Pittsburgh, University of Pittsburgh, Pittsburgh, Pennsylvania, United States of America; National Institutes of Health, UNITED STATES

## Abstract

Prenatal hypoxia is a gestational stressor that can result in developmental abnormalities or physiological reprogramming, and often decreases cellular capacity against secondary stress. When a developing fetus is exposed to hypoxia, blood flow is preferentially redirected to vital organs including the brain and heart over other organs including the kidney. Hypoxia-induced injury can lead to structural malformations in the kidney; however, even in the absence of structural lesions, hypoxia can physiologically reprogram the kidney leading to decreased function or increased susceptibility to injury. Our investigation in mice reveals that while prenatal hypoxia does not affect normal development of the kidneys, it primes the kidneys to have an increased susceptibility to kidney injury later in life. We found that our model does not develop structural abnormalities when prenatally exposed to modest 12% O_2_ as evident by normal histological characterization and gene expression analysis. Further, adult renal structure and function is comparable to mice exposed to ambient oxygen throughout nephrogenesis. However, after induction of kidney injury with a nephrotoxin (cisplatin), the offspring of mice housed in hypoxia exhibit significantly reduced renal function and proximal tubule damage following injury. We conclude that exposure to prenatal hypoxia *in utero* physiologically reprograms the kidneys leading to increased susceptibility to injury later in life.

## Introduction

Intrauterine hypoxia leading to prenatal hypoxia of the fetus is a common gestational stressor that can lead to a range of developmental abnormalities [1–4]. When prenatal hypoxia occurs, the developing fetus exhibits a preferential redirection of blood flow, including oxygen and nutrient supply, to the brain and heart over less vital organs such as the kidney [5, 6]. Previous studies in mouse and rat models of prenatal hypoxia often utilize a chambered hypoxia device, where pregnant dams are housed at 12% O_2_ [7, 8]. These studies show that exposure to modest hypoxia *in utero* can impair pre- and post-natal development [9] and can induce epigenetic modifications [10]. Although both structural and non-structural abnormalities can arise from prenatal exposure to hypoxia, the long-term effect of this stressor in the kidneys is still largely unknown.

The kidneys are the filtration units of the body and the nephron is the functional unit of the kidney responsible for blood filtration, removal of toxic wastes, and regulation of several important physiological functions [11–13]. Kidney development in the mouse takes place from embryonic day 10.5 (E10.5) through postnatal day 3 (P3) corresponding to approximate gestational weeks 5 through 35 in humans [14]. During this time, a population of self-renewing cells called nephron progenitors rapidly proliferate then undergo differentiation to give rise to the glomerular and renal tubular epithelia of the mature nephron [15]. This process of nephron development occurs under physiological hypoxia (~1–9% O_2_) [7], and although temporal changes in oxygen tension are normal, chronic hypoxia can be detrimental [16].

Prenatal hypoxia is a multifactorial condition and can be caused by a variety of factors including placental insufficiency, pre-eclampsia, malnutrition, and even high altitude living during pregnancy [8, 17]. Prenatal hypoxia results in decreased blood and nutrient flow to the fetus leading to decreased oxygen availability in the kidney [5, 6]. In consequence, any complication resulting in prenatal fetal hypoxia can cause changes in fetal growth or organ functionality thereby increasing the risk for diseases later in life [17]. Maternal chronic prenatal hypoxia of less than 10% oxygen has been shown to lead to physical and structural abnormalities [1, 6, 18, 19]. In one study, pregnant dams with a CD1 background housed in 12% O_2_ from E14.5 to P1 found that female offspring were unaffected, however male offspring exhibited decreased body weight, decreased kidney weight, and alterations in tubular development and in the corticomedullary ratio [3]. To our knowledge few studies have used 12% O_2_ conditions and the present investigation is the first to characterize a secondary injury phenotype. A modest 12% O_2_ concentration corresponds to high altitude living (>2,500 meters above sea level) at which more than 140 million people currently reside. Although the oxygen concentration at 2,500 meters above sea level is approximately 15%, people can reside up to approximately 4,500 meters above sea level, which supplies them with around 12% O_2_. The effects of gestational low oxygen conditions on kidney development and long-term kidney function are currently unknown. Although structural abnormalities have been previously shown to arise due to 8–10% oxygen exposure, we hypothesize that even in the absence of structural malformations, the kidneys are more susceptible to disease later in life as a result of physiological reprogramming, a phenomena that decreases cellular functionality of the kidneys to be more susceptible to injury later in life.

One such injury that affects both pediatric and adult patient populations is acute kidney injury (AKI). AKI is an abrupt loss of kidney function characterized by significant decreases in glomerular filtration as well as increases in serum creatinine and blood urea nitrogen (BUN) levels leading to high morbidity and mortality rates [20, 21]. It is also a risk factor for progression to chronic kidney disease (CKD) and eventually end-stage renal disease (ESRD) [22]. It is known that AKI is multifactorial and caused by distinct insults including ischemia, nephrotoxins, and sepsis [23]. Cisplatin is a widely used cytotoxic chemotherapeutic agent and is a known nephrotoxin that causes acute and chronic injury in the kidneys [24, 25]. A major complication of cisplatin treatment is AKI, which occurs in up to 30% of patients [24]. Cisplatin targets both nuclear and mitochondrial DNA and for this reason primarily affects the highly metabolically active proximal tubules [25, 26]. While multiple conditions such as aging and CKD also predispose to AKI [27, 28], there is little known about the effect of prenatal hypoxia on susceptibility to AKI.

Herein, we generated a mouse model of prenatal hypoxia and uncovered a sub-pathological role for hypoxia as a mediator of susceptibility to kidney injury. Our study reveals that mice exposed to prenatal hypoxia during nephrogenesis exhibit exacerbated cisplatin-induced AKI in adulthood. Although we did not observe any malformations or abnormalities in developing or even post-natal kidneys, secondary insult resulted in decreased renal function and more severe injury. Our study highlights the importance of strict regulation of oxygenation in the developing kidney and suggests prenatal hypoxia may physiologically reprogram the nephrons, leaving them more susceptible to injury.

## Methods

### Prenatal hypoxia in mice

Six C57BL/6 wild type time-mated females (gestation age E10.5; Charles River) were placed in a hypoxia chamber. The chamber was equipped with a purge airlock system, CO_2_ and O_2_ control indicators, and humidity and gas monitoring control systems designed for live animal experiments. Exposure to 12% hypoxia was initiated on embryonic day 10.5 (E10.5), which coincides with the induction of nephrogenesis. Hypoxia exposure was terminated on postnatal day 3 (P3), which coincides with the conclusion of nephrogenesis. Six control mice were housed in ambient conditions at 21% O_2_ (normoxia control). For embryonic renal assessments, two pregnant dams were sacrificed from normoxic and hypoxic conditions on E16.5 (normoxia pups N = 4; hypoxia pups N = 4) and two on E18.5 (normoxia pups N = 3; hypoxia pups N = 7) by CO_2_ inhalation followed by cervical dislocation. For post-natal renal assessment, pups from two different dams per condition were transferred to ambient O_2_ on P3 and sacrificed at 7 weeks of age by CO_2_ inhalation followed by cervical dislocation (normoxia pups N = 6; hypoxia pups N = 8). Kidney tissue was immediately collected and stored for analysis. The University of Pittsburgh Institutional Animal Care and Use Committee approved all experiments (Approval No. 16088935).

### Cisplatin-induced AKI in mice

For the secondary stressor model, another cohort of six time-mated female C57BL/6 wild type mice gestation age E10.5 were used following the same normoxia and hypoxia conditions as outlined above from E10.5 through P3. The mice exposed to prenatal hypoxia or normoxia were maintained in regular vivarium with 21% O_2_ ambient conditions until 7 weeks of age. At 7 weeks of age, the male mice were treated with cisplatin (APP NDC 63323-103-64, 20mg/kg bw, ip) in normal saline or with normal saline vehicle control—the effectiveness of this dosage and timing of cisplatin injury was previously confirmed [29]. The mice were sacrificed at peak injury 72 hours after cisplatin treatment by cervical dislocation after isoflurane anesthesia. Blood and kidney tissue were immediately collected and stored for analysis. (Normoxia mice N = 6; hypoxia mice N = 4).

### Tissue collection and histological assessment

Tissue was collected at E16.5 and 7 weeks and immediately flash frozen in liquid nitrogen or fixed in 4% paraformaldehyde (PFA). Fixed tissue was processed and embedded in paraffin. Embedded tissue was sectioned at 4 μm thickness and stained with hematoxylin and eosin (H&E) for histological examination. Renal tubular pathology was semi-quantitvely scored (0: no injury to 5: severe injury) in terms of tubular dilatation and formation of proteinaceous casts. Histological scoring was performed in a blinded fashion at 40x magnification on corticomedullary regions of the tissue sections from N = 4 animals. Eight sections were evaluated for injury per sample. Samples were imaged using a Leica DM 2500 microscope (Leica) and LAS X software (Leica). Glomerular quantification was performed blinded using a low power field. Manual counting was done on the corticomedullary regions of the tissue sections from N = 4 animals. Corpuscle and glomerular quantification was done using ImageJ Software using N = 4 animals and were measured in triplicate and averaged per glomeruli or corpuscle. The glomerular-to-corpuscle ratio was calculated by dividing the average length per glomeruli by the average corpuscle length per corresponding corpuscle.

### Immunofluorescent staining and quantification

Immunofluorescent staining was performed on paraffin embedded samples prepared as described above. Samples were probed using primary antibodies or lectins (1:100) against: neural cell adhesion molecule (Ncam; Sigma-Aldrich), sine oculis homeobox homolog 2 (Six2; Proteintech), phospho-histone H3 (pHH3; Cell Marque), endomucin (Santa Cruz Biotechnology), Wilm’s tumor 1 (WT1; Santa Cruz Biotechnology), lotus tetragonolobus (LTL; Vector Laboratories), organic anion transporter 1 (OAT1; Alpha Diagnostic International), dolichos biflorus agglutinin (DBA; Vector Laboratories), kidney injury molecule 1 (Kim1; R&D Systems), and terminal deoxynucleotidyl transferase dUTP nick-end labeling kit (TUNEL; EDM Millipore) all co-stained with DAPI (Thermo Fisher Scientific). Samples were imaged using a Leica microscope (Leica Microsystems) and LAS X software (Leica Microsystems). Quantification of proximal tubule dilation was done using ImageJ Software. Quantification was done in triplicate based on the average of five proximal tubules per animal (N = 3 animals). Quantification of Kim 1 intensity was done using ImageJ Software.

### Real time quantitative polymerase chain reaction (RT-qPCR)

RNA was extracted from the flash frozen kidney tissue using the RNeasy Mini Kit (Qiagen). Superscript First Strand cDNA kit (Invitrogen, CA) was used for cDNA synthesis. The cDNA from these samples was analyzed using a C1000 Thermal Cycler (Bio-Rad, Hercules, CA) to determine the levels of mRNA in the tissue. The following genes were analyzed using this method: *Cited1*, *Wnt4*, *Lhx1*, and *Pax2*. Gene expression was normalized to *Rn18s*. Primer sequences are listed in S1 Table.

### Serum analysis

At 7 weeks of age or after cisplatin injection, animals were subjected to cardiac punctures prior to sacrifice for the collection of blood. Blood was subsequently centrifuged and serum was collected. Serum was analyzed for blood urea nitrogen and creatinine at the Kansas State Veterinary Diagnostic Laboratory.

### Statistical analyses

Three or more biological replicates were used for each experiment outlined. When comparing two sample groups, statistical significance was determined using a two-tailed Student’s *t* test (α = 0.05). Data is presented as standard error of the mean (SEM). Histology and immunofluorescent images were generated using Photoshop and quantified using ImageJ. Graphs were generated using GraphPad Prism.

## Results

### Prenatal hypoxia does not alter embryonic kidney development

**Fig 1:**
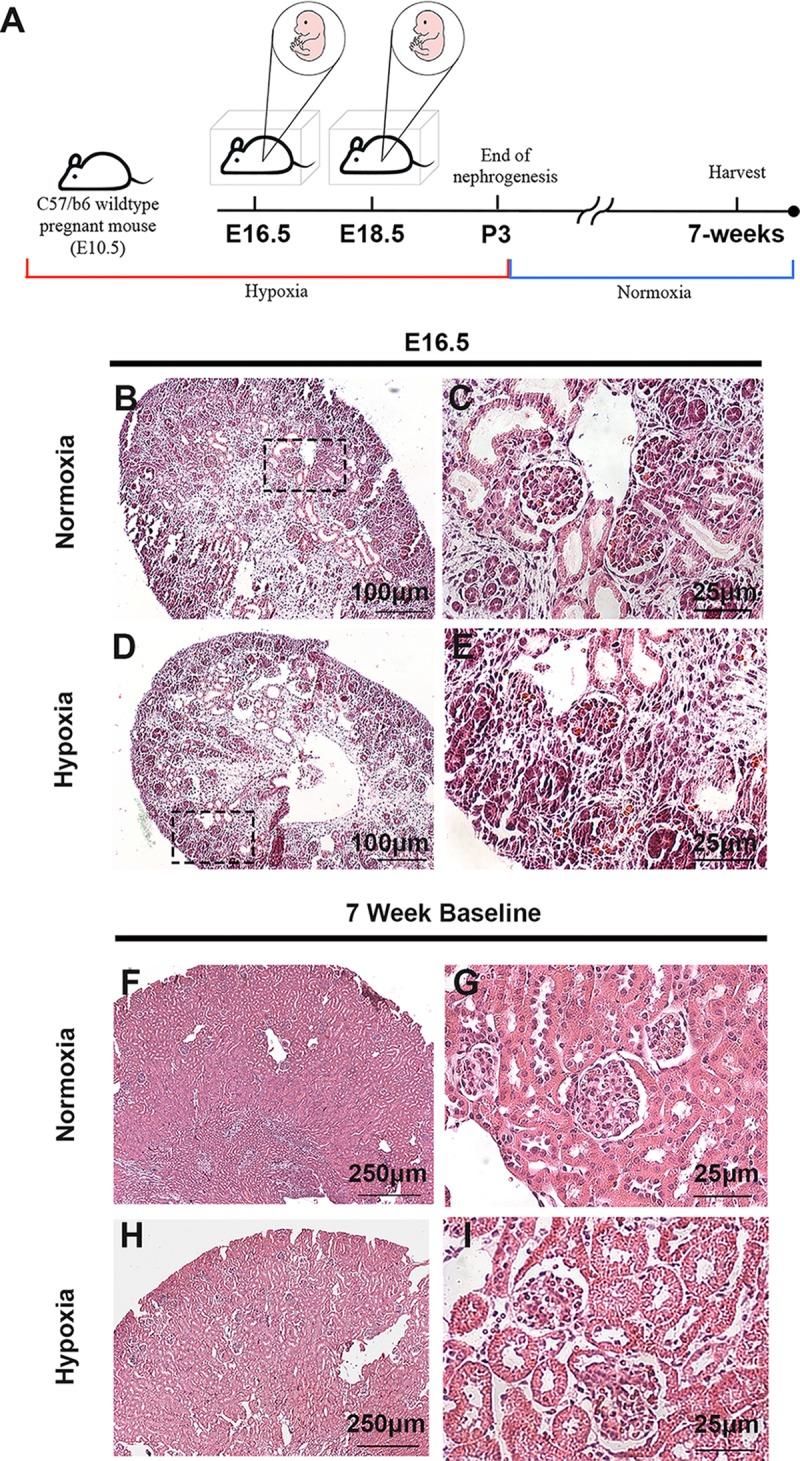
Prenatal hypoxia does not alter histological structure of the kidneys. (**A**) Schematic illustration for the mouse model of prenatal exposure to hypoxia during kidney development. (**B-E**) H&E staining from E16.5 prenatal normoxia and hypoxia exposed kidneys. (**F-I**) H&E staining from 7-week mice exposed to prenatal normoxia and hypoxia.

**Fig 2:**
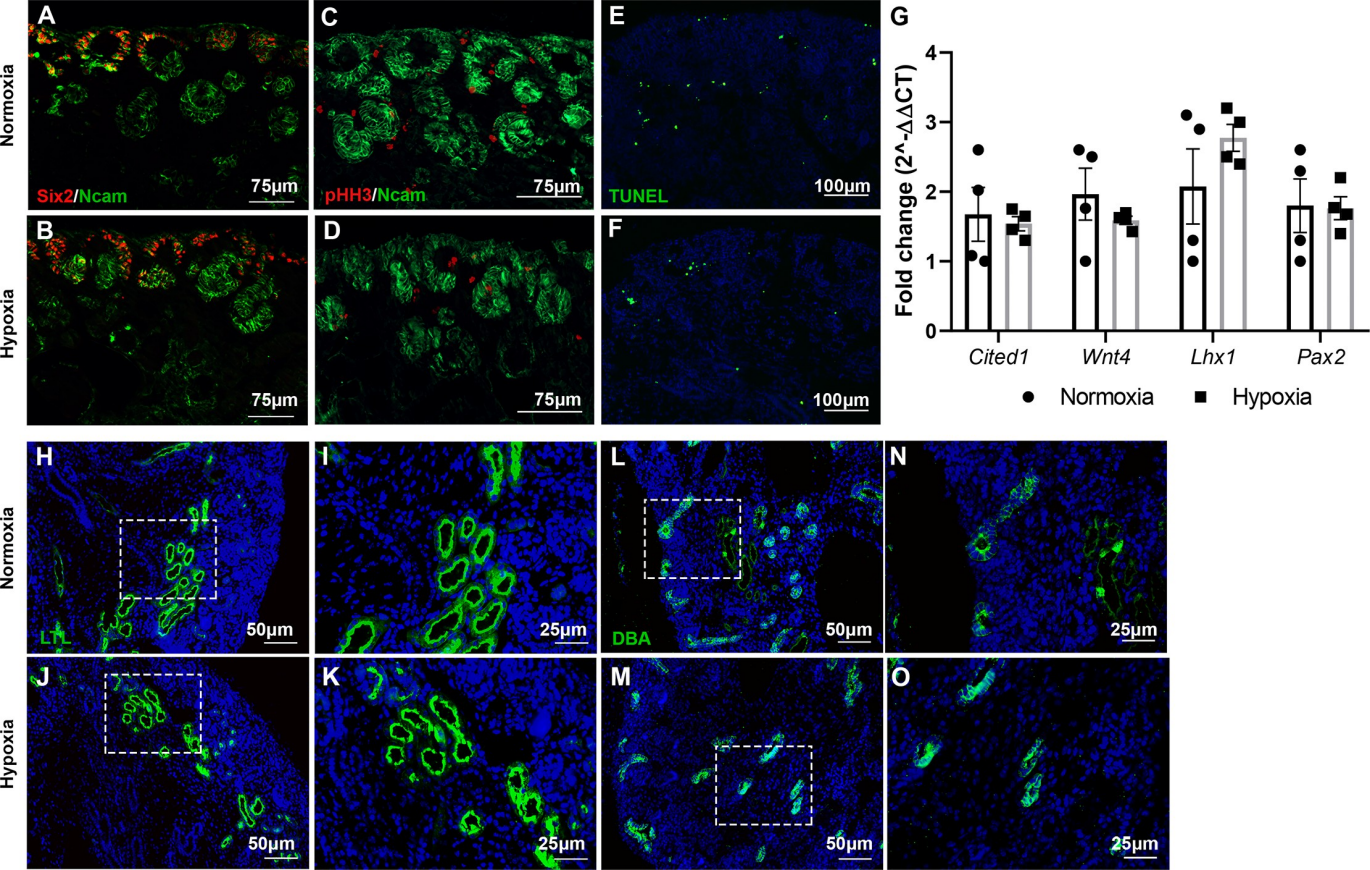
Prenatal hypoxia does not alter normal development of the kidneys at E16.5. (**A-B**) Immunofluorescent staining with antibodies against Six2 (red) and Ncam (green) to show nephron progenitors and nascent nephrons from prenatal normoxia (top) versus hypoxia (bottom) kidneys at E16.5. (**C-D**) Immunofluorescent staining with antibodies against pHH3 (red) and Ncam (green) in E16.5 kidneys exposed to prenatal normoxia (top) versus hypoxia (bottom) to determine the number of mitotic cells. (**E-F**) TUNEL (green) staining of E16.5 kidneys in prenatal normoxic (top) versus hypoxic cells (bottom) showing representative apoptotic cells. (**G**) RT-qPCR analysis of genes associated with nephron progenitors (*Cited1*, *Pax2*) and differentiation (*Wnt4* and *Lhx1*) between prenatal normoxia- (black) and hypoxia- (grey) exposed E16.5 kidneys. N = 4. Error bars indicated as SEM. (**H-K**) Immunofluorescent staining with LTL to monitor proximal tubule development at E16.5 between prenatally-exposed normoxic (top) and hypoxic (bottom) kidneys. (**L-O**) Immunofluorescent staining against DBA to monitor ureteric bud branching and differentiation at E16.5 in normoxia (top) and hypoxia (bottom) exposed kidneys.

To investigate the effect of hypoxia during kidney development, time-mated C57B/6 female mice were placed into hypoxia chambers with 12% oxygen beginning on embryonic day 10.5 (E10.5; start of nephrogenesis) and maintained in hypoxia until the conclusion of nephrogenesis on postnatal day 3 (P3) (Fig 1A). Pregnant control females were housed in ambient oxygen (normoxia) for the duration of their pregnancy. Litter sizes between dams housed in normoxia and hypoxia were similar (P = 0.67). Hypoxia exposed E16.5 (mid point of nephrogenesis) kidneys appeared to undergo normal developmental processes and histological examination did not reveal any significant abnormalities (Fig 1B–1E). A cohort of pups exposed to prenatal hypoxia were transferred to normoxia at P3 and at 7 weeks of age those mice also did not exhibit any significant alterations in renal histology (Fig 1F–1I). To further investigate whether any developmental defects were present, we performed immunofluorescent staining on E16.5 kidneys to evaluate kidney development using markers for nephron progenitor self-renewal (Sine oculis homeobox homolog 2; Six2) and developing structures (Neural cell adhesion molecule; Ncam). Neither Six2 nor Ncam immunostaining revealed any structural malformations or alterations in nephron number. Proliferation of the nephron progenitors was evaluated by immunostaining against phospho-histone H3 (pHH3), which appeared to be occurring at comparable rates in both normoxia and hypoxia-exposed kidneys. Apoptosis analysis through TUNEL immunostaining showed that cell death was confined to the renal stroma [30] and appeared comparable between normoxia and hypoxia exposed kidneys (Fig 2A–2F). Additionally, mRNA levels of nephron progenitors (*Cited1* and *Pax2*) and nephron differentiation (*Wnt4* and *Lhx1*) were not altered between the two cohorts (Fig 2G). Likewise, we performed immunostaining to observe proximal tubule development and ureteric branching, neither of which revealed any abnormalities (Fig 2H–2O). Together, this suggests that prenatal exposure to 12% oxygen in the C57B/6 background strain does not lead to structural alterations in kidney development by mid-nephrogenesis.

**Fig 3:**
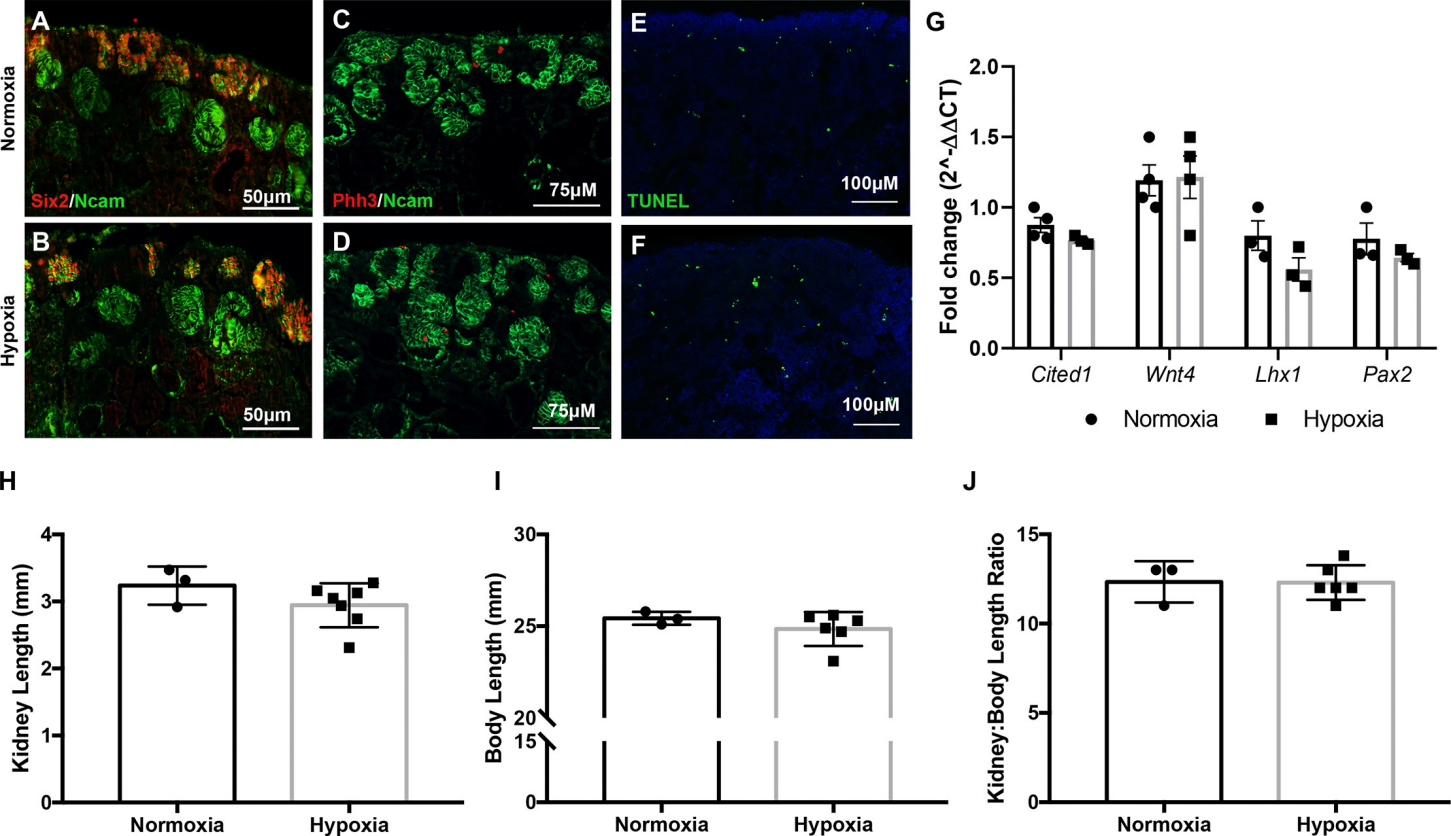
Prenatal hypoxia does not alter normal development of the kidneys at E18.5. **(A-B)** Immunofluorescent staining with antibodies against Six2 (red) and Ncam (green) to mark nephron progenitors and nascent nephrons in kidneys exposed to prenatal normoxia (top) versus hypoxia (bottom) at E18.5. **(C-D)** Immunofluorescent staining with antibodies against pHH3 (red) and Ncam (green) to monitor proliferation in normoxia (top) and hypoxia (bottom) exposed kidneys at E18.5. **(E-F)** TUNEL (green) staining showing representative apoptosis in prenatally-exposed normoxic (top) and hypoxia cells (bottom) from E18.5 kidneys. **(G)** RT-qPCR analysis of genes associated with nephron progenitors (*Cited1*, *Pax2*) and differentiation (*Wnt4* and *Lhx1*) between prenatal normoxia- (black) and hypoxia- (grey) exposed E18.5 kidneys. N = 3. Error bars indicated as SEM. **(H-J)** Quantification of kidney length, body length, and the kidney-to-body length ratio of E18.5 embryos. Normoxia N = 3; Hypoxia N = 6–7. Error bars indicated as SEM.

We next evaluated the progression of renal development at E18.5—a late stage of kidney development. Similarly, we immunostained for nephron progenitors (Six2, Ncam), differentiated structures (Ncam), proliferation (pHH3), and apoptosis (TUNEL) and did not observe any abnormalities at this stage of development (Fig 3A–3F). Gene expression of nephron progenitor (*Cited1*, *Pax2)*, and differentiation markers (*Wnt4*, and *Lhx1)* also remained unchanged between the two groups (Fig 3G). Lastly, we quantified the kidney length and body length of E18.5 animals and did not reveal any differences in the size of the kidneys or bodies of offspring exposed to hypoxia (Fig 3H and 3I). The kidney-to-body length ration also remained consistent (Fig 3J). Therefore, we did not reveal any structural differences during kidney development in mice exposed to prenatal hypoxia.

### Prenatal hypoxia alone does not alter renal function in the adult

**Fig 4:**
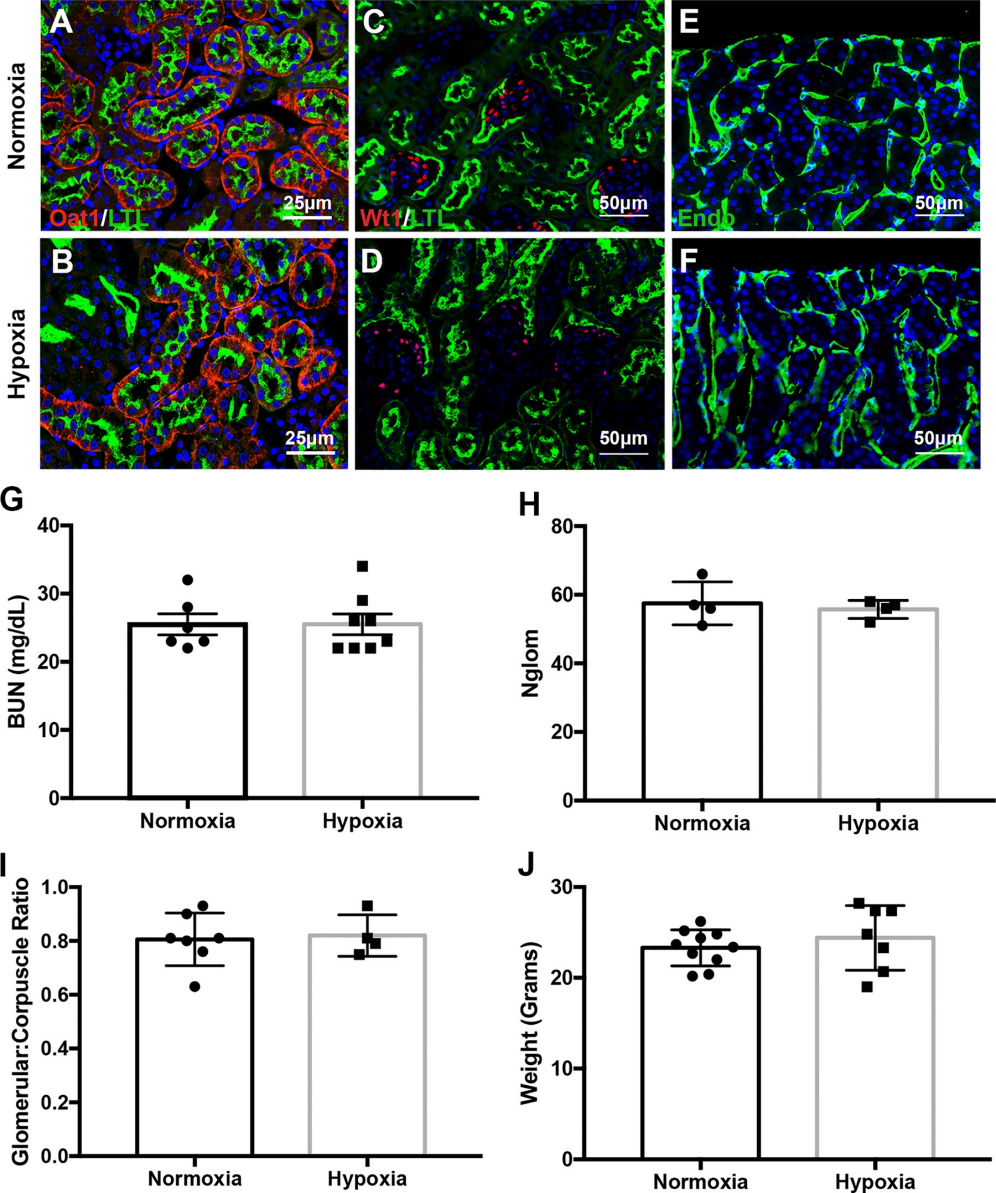
Prenatal hypoxia does not alter the structure or function of 7 week kidneys. (**A-B**) Immunofluorescent staining with antibodies against Oat1 (red) and LTL (green) denoting the proximal tubules of prenatal hypoxia-exposed kidneys (bottom) when compared to normoxia-exposed kidneys (top) at 7 weeks. (**C-D**) Immunofluorescence staining with antibodies against Wt1 (red) and LTL (green) showing podocytes represent the glomerular structure at 7 weeks in normoxia and hypoxia exposed kidneys. (**E-F**) Immunofluorescence staining with antibodies against endomucin (Endo; green) indicating the level of vascularization in kidneys from animals exposed to normoxia or hyoxpia. (**G**) Quantification of renal function by measurement of blood urea nitrogen (BUN) in 7-week normoxia and hypoxia exposed animals; Normoxia N = 6; Hypoxia N = 7; Error bars indicated as SEM. (**H**) Quantification of glomerular number indicative of nephron quantity in normoxia versus hypoxia exposed kidneys at 7-weeks. N = 4. Error bars indicated as SEM. (**I**) Quantification of the size of the glomeruli and corpuscles of animals exposed to prenatal hypoxia compared to controls as indicated by the ratio of glomerular to corpuscle size; Normoxia N = 7; Hypoxia N = 4; Error bars indicated as SEM. (**J**) Quantification of the body weight of animals exposed to prenatal hypoxia compared to controls; Normoxia N = 10; Hypoxia N = 7; Error bars indicated as SEM.

Next, we assessed renal structure and function at 7 weeks of age, this age was selected as nephrogenesis is complete but no injury should be observed in wild type kidneys at this time. Immunofluorescent staining using LTL and Oat1 (markers of proximal tubule brush borders and proximal tubule epithelial cells, respectively) did not reveal the development of structural abnormalities by 7-weeks of age (Fig 4A and 4B). There were also no significant differences in glomerular structure (Fig 4C and 4D) or vascularization (Fig 4E and 4F) in 7-week mice exposed to prenatal hypoxia compared to normoxia-exposed controls. BUN measurements were also not significantly altered in prenatal hypoxia exposed mice (Fig 4G). Creatinine levels were all below a detectable 0.5 mg/dL concentration. Therefore, prenatal hypoxia does not seem to influence kidney function by 7 weeks of age. To confirm that nephrogenesis occurred normally, we quantified the number of glomeruli using an unbiased manual counting approach of the glomerular structures. Both mouse cohorts had similar numbers of formed glomeruli (Fig 4H). To confirm that proximal tubule development and subsequent function appeared normal, we quantified gene expression of the outer mitochondrial membrane gene *Tomm20*. No differences in Tomm20 expression was observed between the two conditions (S1 Fig). Further, we quantified the corpuscle and glomerular size as shown by the glomerular-to-corpuscle ratio, which again did not reveal any differences in nephron development (Fig 4I). Lastly, the weight of the animals did not differ between exposure groups (Fig 4J). Therefore, mild prenatal hypoxia alone does not lead to alterations in renal structure or function in C57B/6 adult mouse kidneys.

### Prenatal hypoxia increases susceptibility to cisplatin-induced AKI

**Fig 5:**
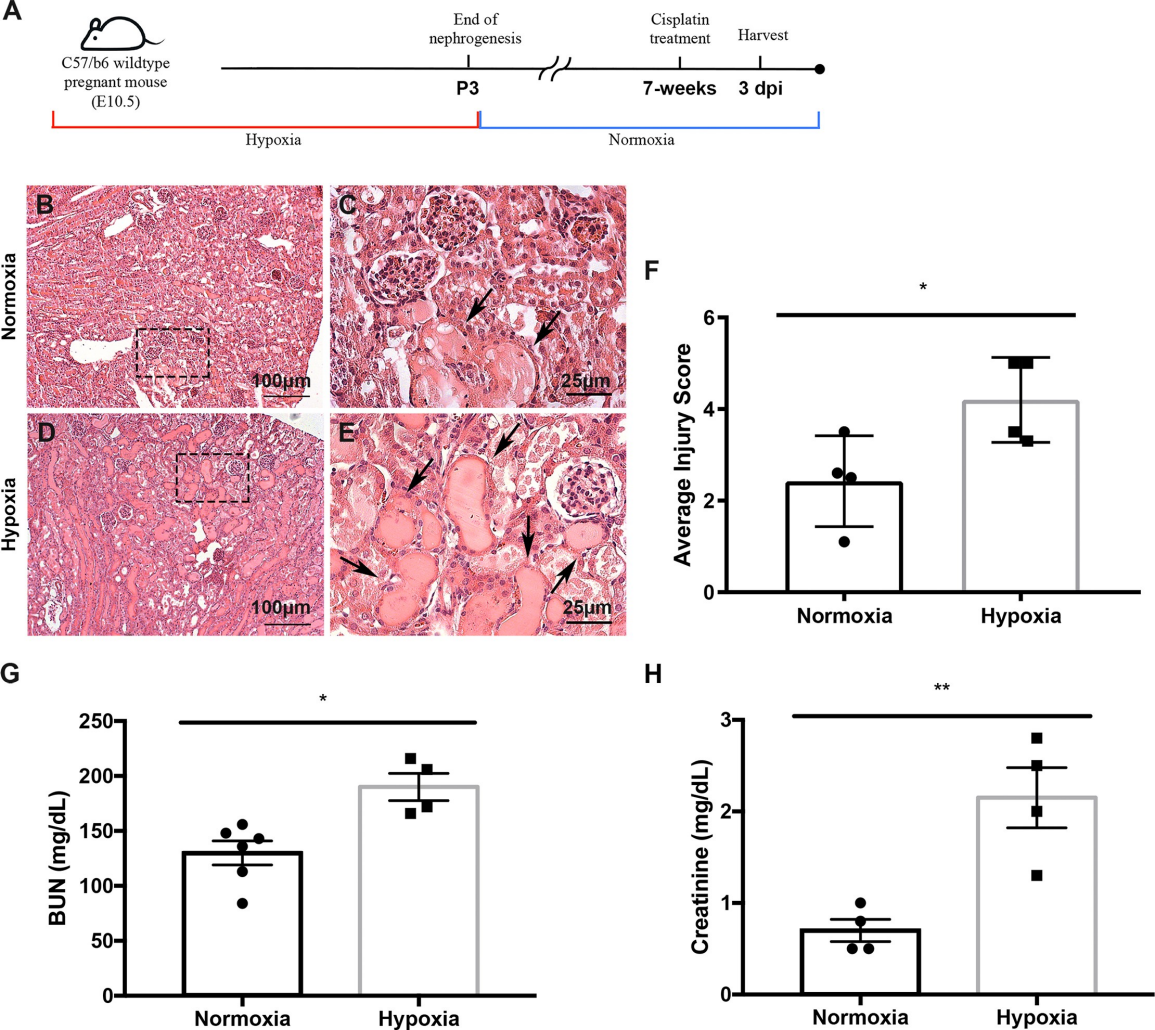
Prenatal hypoxia exposure decreases renal function after cisplatin-induced AKI. (**A**) Schematic illustration for mouse model of prenatal hypoxia, coupled with cisplatin-induced AKI in adult mice exposed to normoxia or hypoxia during development. (**B-E**) H&E staining of kidneys exposed to prenatal hypoxia and treated with cisplatin at 7 weeks (bottom panels) depicting formation of proteinaceous casts (arrows) and dilation of the proximal tubules in kidneys exposed to prenatal normoxia with cisplatin treated kidneys (top panels). (**F**) Quantification of the severity of injury in the kidneys of mice exposed to prenatal normoxia or hypoxia; P<0.05, N = 4. (**G**) Quantification of renal function by blood-urea nitrogen measurement in mice exposed to prenatal normoxia or hypoxia and treated with cisplatin at 7-weeks; *P<0.05; Normoxia N = 7 Hypoxia N = 4. Error bars indicated as SEM. (**H**) Quantification of renal function by creatinine measurement in mice exposed to prenatal normoxia or hypoxia and treated with cisplatin at 7-weeks; **P<0.01; N = 4. Error bars indicated as SEM.

**Fig 6:**
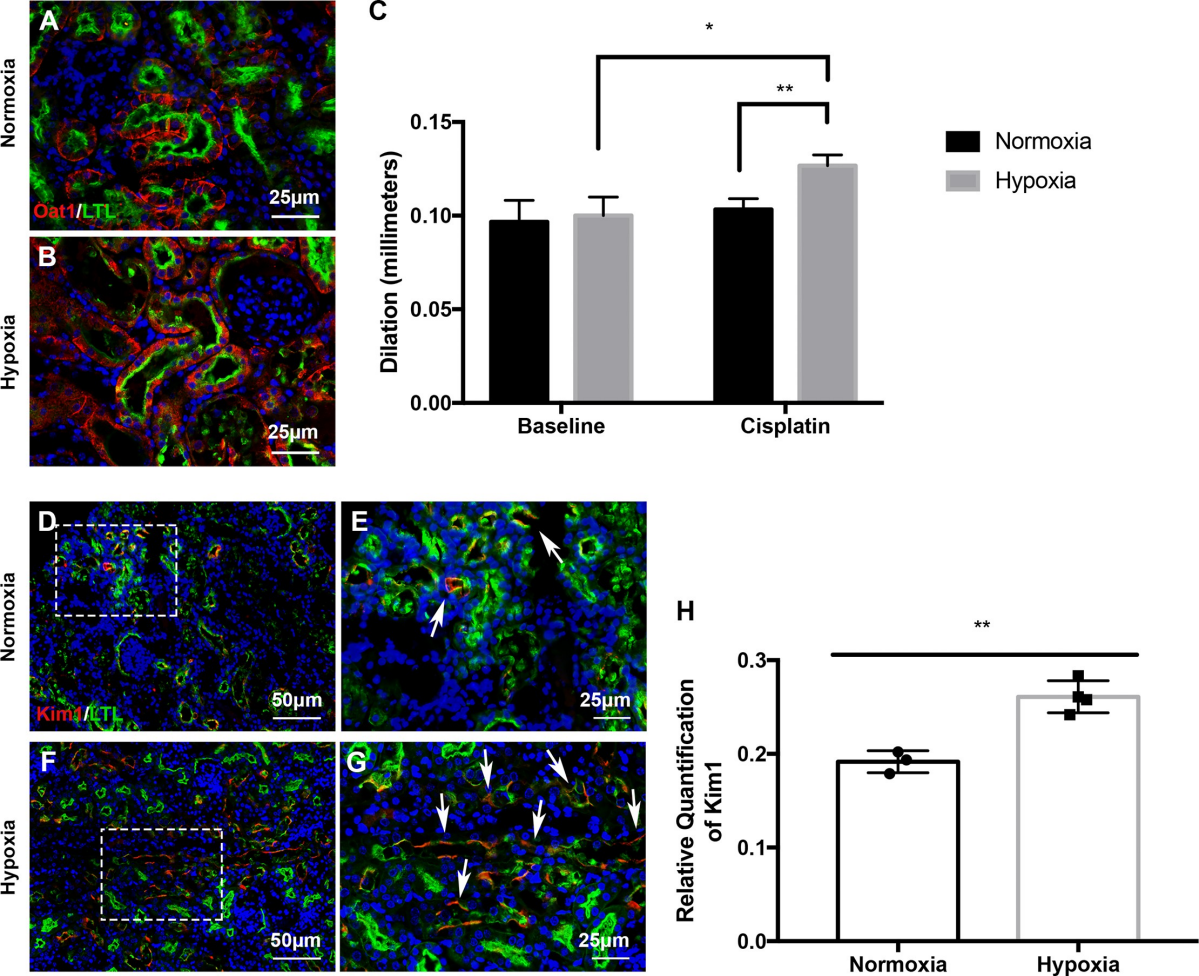
Prenatal hypoxia exposure increases proximal tubular injury after cisplatin-induced AKI. (**A-B**) Immunofluorescence staining with antibodies against Oat1 (red) and LTL (green) after 3 dpi indicating proximal tubule health in prenatal hypoxia exposed mice after cisplatin treatment. (**C**) Quantification of proximal tubule dilation in mice that were untreated and 3dpi; *P<0.05, P<0.01; N = 4; Error bars indicated as SEM. (**D-G**) Immunostaining with antibodies against Kim1 (red) and LTL (green) at 7-weeks to monitor renal injury (arrows) in prenatal normoxia and hypoxia-exposed kidneys (bottom) treated with cisplatin. (**H**) Quantification of the Kim1-positive area in prenatal normoxia and hypoxia exposed kidneys; **P<0.01, Normoxia N = 3; Hypoxia N = 4. Error bars indicated as SEM.

Next, we aimed to determine whether prenatal hypoxia in the absence of structural abnormalities led to increased kidney injury susceptibility. Another cohort of mice that were exposed to prenatal hypoxia were subjected to a single injection of the nephrotoxin cisplatin (20mg/kg bw, ip) and were sacrificed 3 days post injury (3 dpi) (Fig 5A). The prenatal hypoxia kidneys exhibited exacerbated cisplatin-induced injury. Injury included increased formation of proteinaceous casts and severe dilation of the proximal tubules (Fig 5B–5E) when compared to normoxia-exposed kidneys. Histopathological scoring revealed a significantly higher level of injury severity (P = 0.038; Fig 5F). The prenatally hypoxia-exposed kidneys also had reduced Tomm20 expression as evidenced by immunofluorescent staining signifying enhanced tubular injury (S1 Fig). Accompanying the tubular injury, these mice exhibited significantly increased levels of blood urea nitrogen (BUN) and creatinine in serum indicating reduced kidney function (Fig 5G and 5H). Furthermore in the prenatal hypoxia exposed kidneys, immunofluorescent staining with LTL and Oat1 revealed increased frequency of epithelial sloughing and dilation in the proximal tubules (Fig 6A and 6B). Proximal tubule dilation was quantified and revealed prenatal hypoxia-exposed kidneys treated with cisplatin had significantly increased dilation compared to both untreated 7-week hypoxia and cisplatin-treated normoxia-exposed kidneys (Fig 6C). Hypoxia-exposed kidneys treated with cisplatin also had significantly increased expression of kidney injury marker 1 (Kim1) (Fig 6D–6H). With the severity of the injury and potential for physiological reprogramming, we evaluated the expression known hypoxia inducible factor (Hifs) target genes including *NADPH oxidase 4 (Nox4)*, *BCL2 interacting protein 3 (Bnip3)*, *and glyceraldehyde-3-phosphate dehydrogenase (Gapdh)*. We found that although these genes were relatively unchanged during development and at 7-weeks, after cisplatin injury Hif-associated gene expression significantly increased (S2 Fig) indicating that exposure to hypoxia *in utero* likely plays a role in increasing susceptibility to a secondary insult such as cisplatin later in life.

## Discussion

Our study did not reveal any structural developmental kidney defects in offspring of dams exposed to hypoxia. At both 12% O_2_ and ambient oxygen (~21% O_2_), the kidneys exhibited a comparable degree of differentiation, proliferation and apoptosis of E16.5 and E18.5 nephron progenitors regardless of oxygen treatment. Although this finding differs slightly from previous investigations utilizing similar (8–10% O_2_) hypoxia conditions [4, 31, 32], we hypothesize that the lack of developmental defect could be explained by the difference in actual oxygen concentration and by the mouse strain used in previous studies. Our study used a modest hypoxia model with an oxygen concentration set at 12% O_2_ to maximize survival by treatment and to mimic high altitude living. Other studies often use a more severe oxygen concentrations. One study using 12% O_2_ and mice with a CD-1 background showed that 12% O_2_ was sufficient to mediate structural abnormalities during development, however our investigation utilized mice with a C57B/6 background. The CD-1 mouse strain also exhibits higher mortality in response to hypoxia [33] by activating a maladaptive hypoxia response through Hif-1α up regulation [34]. Therefore, this is a plausible reason behind the lack of structural abnormalities in our model, thus allowing for an interrogation of physiological reprogramming in the absence of structural abnormalities.

Less is currently known about the sub-pathological role of prenatal hypoxia on organ injury later in life. It has previously been shown that prenatal hypoxia decreases adaptive potential of the brain and subsequently increases the risk of neurodegenerative disorders such as Parkinson’s disease in late adulthood [35, 36]. Further the heart has been shown to undergo physiological reprogramming in relation to *in utero* hypoxia leading to injury susceptibility later in life [37–39]. However, prenatal hypoxia in the absence of structural defects has not been well studied in kidney injury models.

To precisely dissect the effect from prenatal hypoxia in susceptibility to kidney disease later in life, we challenged the offspring mice that were exposed to prenatal hypoxia with acute nephrotoxicity via a high dose of cisplatin treatment. Mice subjected to prenatal hypoxia displayed increased susceptibility to cisplatin injury despite the absence of clear developmental defects. Cisplatin causes cellular damages in the S3 segment of renal proximal tubular epithelial cells in mice [40]. Our data demonstrated that cisplatin treatment increased expression of proximal tubule specific injury marker, Kim-1, in the group exposed to prenatal hypoxia as well as increased renal functional markers, creatinine and BUN. This study acts as a proof of principle by clearly showing that strict regulation of developmental oxygenation is critical for long-term renal function. Although prenatal hypoxia exposure leads to exacerbated kidney injury, the mechanism leading to this finding is still unclear, but future investigation into the many influential pathways would be beneficial to the field.

The present study does have limitations and raises a number of questions. It has been previously proposed that age and sex play a role in hypertension pathology after prenatal hypoxia [2]. For this reason, it will be important to evaluate the role of age and sex in susceptibility to cisplatin injury of the offspring after prenatal hypoxia. Additionally, the present mechanism of injury exacerbation is unknown. We explored several potential pathways to explain the increased susceptibility to injury of the prenatal exposed animals including expression of the pro-inflammatory cytokine tumor necrosis factor alpha (*Tnf*), which was not significantly altered between normoxia and hypoxia exposed kidney that were treated with cisplatin (S4 Fig). We also evaluated the expression of genes regulated by Hifs including mitochondrial *Bnip3*, glycolytic *Gapdh*, and reactive oxygen species (ROS) associated *Nox4*. Previously, it was shown that *Nox4* in particular promotes cisplatin-induced AKI through increased ROS [41]. Further, ROS-mediated stress in general has been implicated in the severity of cisplatin-induced kidney injury. Therefore, it is possible that prenatal exposure to hypoxia may have primed the kidneys to be more vulnerable to injury later in life. Investigations into the signaling of Hifs and ROS are complex and in the current literature it is unclear whether ROS promotes cellular survival during injury or leads to worsened outcomes [42–45]. Since our data did not indicate that Hif expression was significantly increased during development (the time of hypoxia exposure), physiological reprogramming may also, in part, be due to epigenetic regulation by Hif-induced post-translational modifications and could be a future avenue of study. Other mechanisms such as Kim-1 induction of ERK/MAPK/STAT3 and dysregulation of the Renin-Angiotensin System (RAS) would also be interesting candidate pathways for future exploration. Future investigations into the effect of developmental hypoxia on long-term renal function should be aimed at providing mechanistic insight that could enhance preventative medicine in high-altitude residents.

In conclusion, we show that embryonic hypoxia exposure predisposes mice to enhanced kidney injury after cisplatin treatment during adulthood even in the absence of initial structural abnormalities. This study suggests that sub-pathologically low oxygen concentrations during fetal development may play a critical role in the physiological programming of the nephron in the absence of structural abnormalities and could be used as a prognosticator for kidney disease susceptibility in adults.

## Supporting information

S1 Table List of primers for real time quantitative PCR. These primers were used for the following genes: *Cited1*, *Wnt4*, *Lhx1*, *Pax2*, *Tomm20*, *Nox4*, *Bnip3*, *Gapdh*, *Tnf*, and *Rn18s*.
(DOCX)

S1 Fig Mitochondrial density is decreased after cisplatin injury. A) *Tomm20* gene expression in E18.5 kidneys is unchanged between animals exposed to prenatal normoxia or hypoxia; P = 0.41; N = 4v4. B) *Tomm20* gene expression in 7-week kidneys is not significantly different in animals exposed to prenatal normoxia or hypoxia (P = 0.32; N = 3v3). C-H) Immunofluorescent staining again Tomm20 (red), LTL (green), and DAPI (blue) in 7-week kidneys treated with cisplatin reveals decreased expression of Tomm20 in animals exposed to prenatal hypoxia.
(TIF)

S2 Fig Hif-target genes are up regulated after cisplatin injury. A) *Nox4* (P = 0.57), *Bnip3* (P = 0.72), and *Gapdh* (P = 0.12) gene expression in E18.5 kidneys is unchanged between animals exposed to prenatal normoxia or hypoxia; N = 4v4. B) *Nox4* (P = 0.81), *Bnip3* (P = 0.52), and *Gapdh* (P = 0.64) gene expression in 7-week kidneys is not significantly different in animals exposed to prenatal normoxia or hypoxia; N = 3v3. C) *Nox4* (P = 0.067), *Bnip3* (P = 0.004), and *Gapdh* (P = 0.0008) gene expression after cisplatin treatment is increased in mice exposed to prenatal hypoxia; N = 3v4.
(TIF)

S3 Fig There is no change is the inflammatory response after cisplatin treatment. A) *Tnf* (P = 0.72) gene expression in 7-week kidneys after cisplatin treatment is unchanged between animals exposed to prenatal normoxia or hypoxia; N = 3v4.
(TIF)

S4 Fig There are not significant differences in apoptosis after cisplatin injury. A-B) TUNEL staining on kidneys after cisplatin treatment shows that there are not differences in the number of apoptotic nuclei after injury.
(TIF)
