# Supplementary material for: Prenatal hypoxia increases susceptibility to kidney injury
Source: PLoS One. 2020 Feb 21;15(2):e0229618. doi: 10.1371/journal.pone.0229618 (PMC7034911; doi:10.1371/journal.pone.0229618)
Supplement: S1 Table — These primers were used for the following genes: Cited1, Wnt4, Lhx1, Pax2, Tomm20, Nox4, Bnip3, Gapdh, Tnf, and Rn18s. (DOCX) [file pone.0229618.s001.docx]

| **Gene** | **Forward** | **Reverse** |
| --- | --- | --- |
| *Cited1* | CCAACCAGGAGATGAACT | AGAGCCTATTGGAGATGTC |
| *Wnt4* | TCTCTGCTCATTGTCCAT | TGCTGAACTAAGTCTACCA |
| *Lhx1* | CTACATCATAGACGAGAACAAG | TCATTACTACCACCTTCCTTAT |
| *Pax2* | GCTAAGGAAAGGACTTTGTG | TAGGCAGTTCAGGTGGAT |
| *Tomm20* | TGGGCTTTCCAAGTTACCTGAT | GGTCGGAAGCTTGGTCAGAA |
| *Nox4* | TCAAACAGCTGTGCTATGCC | ATCAACAGCGTGCGTCTAAC |
| *Bnip3* | TTTAAACACCCGAAGCGCAC | CCAATGTAGATCCCCAAGCCA |
| *Gapdh* | AACCTGCCAAGTATGATGA | GGAGTTGCTGTTGAAGTC |
| *Tnf-α* | TCGTAGCAAACCACCAAGTG | AGATAGCAAATCGGCTGACG |
| *Rn18s* | GACAGGATTGACAGATTGATAG | CCAGAGTCTCGTTCGTTA |

**Supplementary Table 1: List of primers for real time quantitative PCR**. These primers were used for the following genes: *Cited1, Wnt4, Lhx1, Pax2, Tomm20, Nox4, Bnip3, Gapdh, Tnf,* and *Rn18s.*
