# Supplementary figures and images for: Prenatal hypoxia increases susceptibility to kidney injury
Source: PLoS One. 2020 Feb 21;15(2):e0229618. doi: 10.1371/journal.pone.0229618 (PMC7034911; doi:10.1371/journal.pone.0229618)

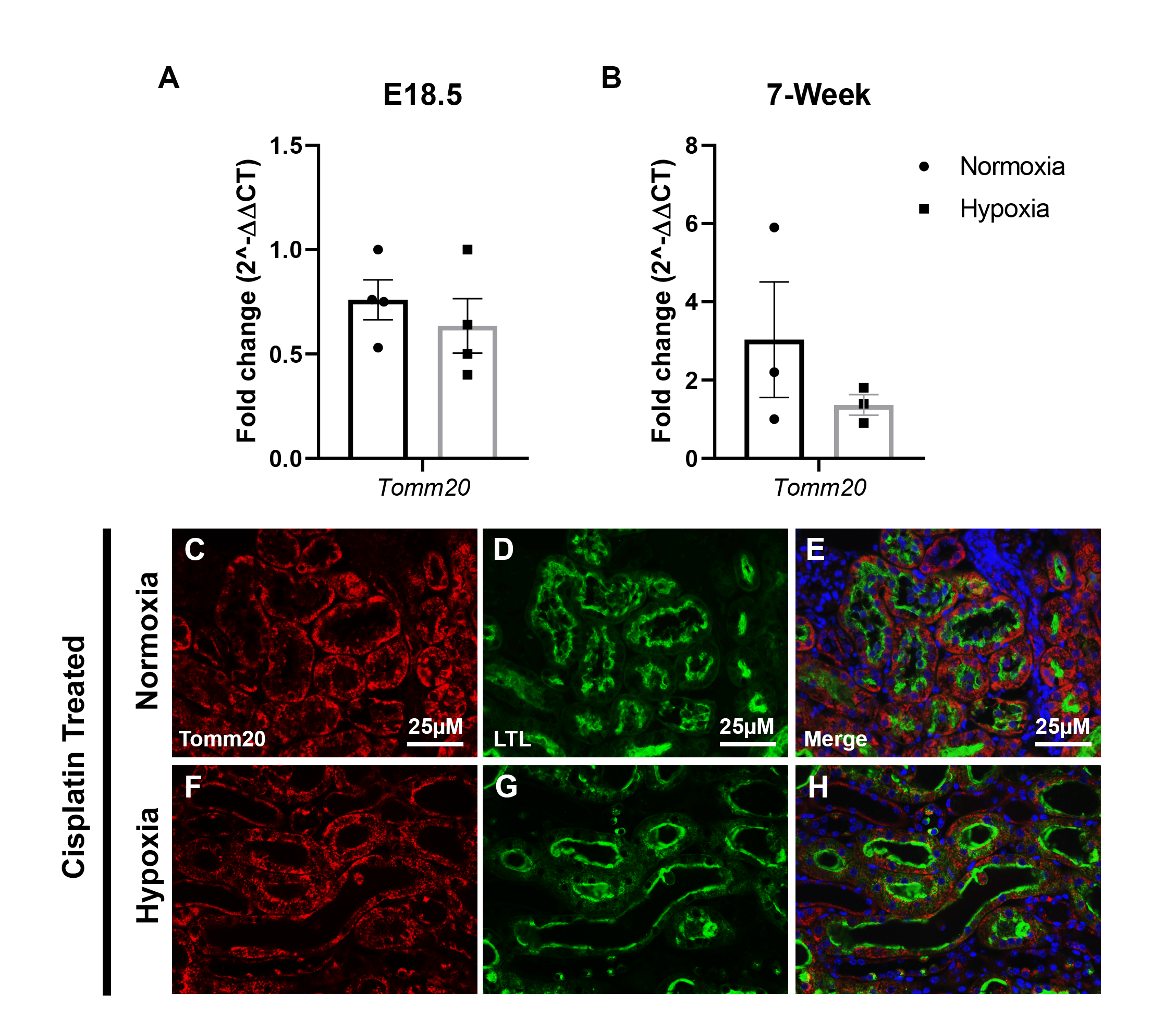

Supplement: S1 Fig — A) Tomm20 gene expression in E18.5 kidneys is unchanged between animals exposed to prenatal normoxia or hypoxia; P = 0.41; N = 4v4. B) Tomm20 gene expression in 7-week kidneys is not significantly different in animals exposed to prenatal normoxia or hypoxia (P = 0.32; N = 3v3). C-H) Immunofluorescent staining again Tomm20 (red), LTL (green), and DAPI (blue) in 7-week kidneys treated with cisplatin reveals decreased expression of Tomm20 in animals exposed to prenatal hypoxia. (TIF) [file pone.0229618.s002.tif]

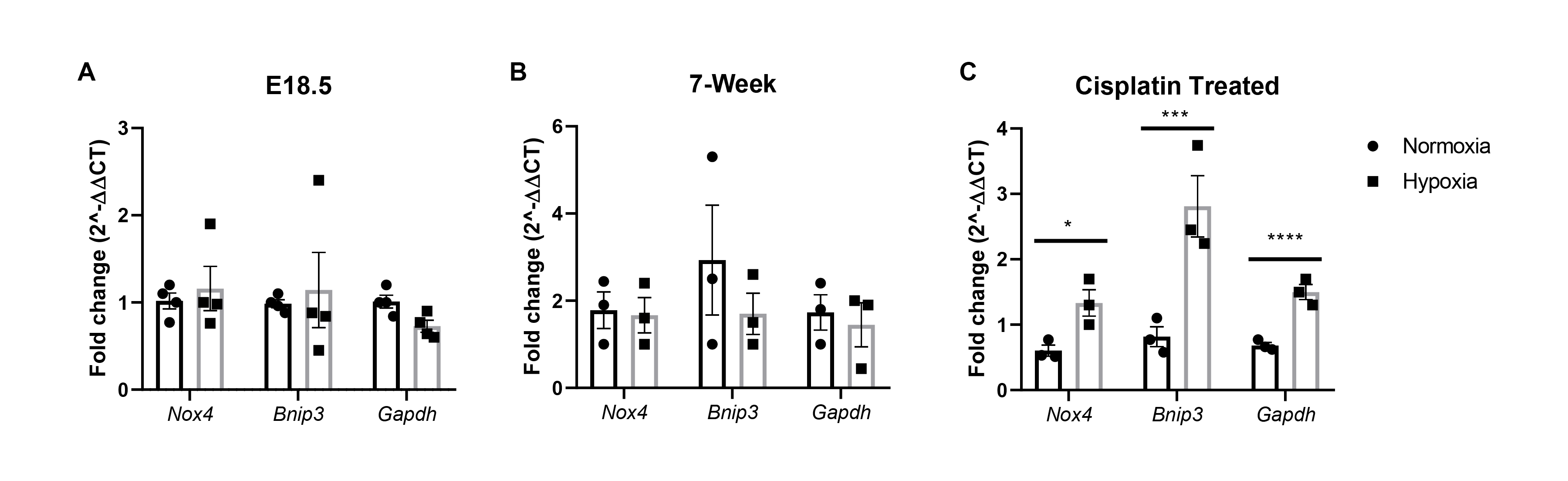

Supplement: S2 Fig — A) Nox4 (P = 0.57), Bnip3 (P = 0.72), and Gapdh (P = 0.12) gene expression in E18.5 kidneys is unchanged between animals exposed to prenatal normoxia or hypoxia; N = 4v4. B) Nox4 (P = 0.81), Bnip3 (P = 0.52), and Gapdh (P = 0.64) gene expression in 7-week kidneys is not significantly different in animals exposed to prenatal normoxia or hypoxia; N = 3v3. C) Nox4 (P = 0.067), Bnip3 (P = 0.004), and Gapdh (P = 0.0008) gene expression after cisplatin treatment is increased in mice exposed to prenatal hypoxia; N = 3v4. (TIF) [file pone.0229618.s003.tif]

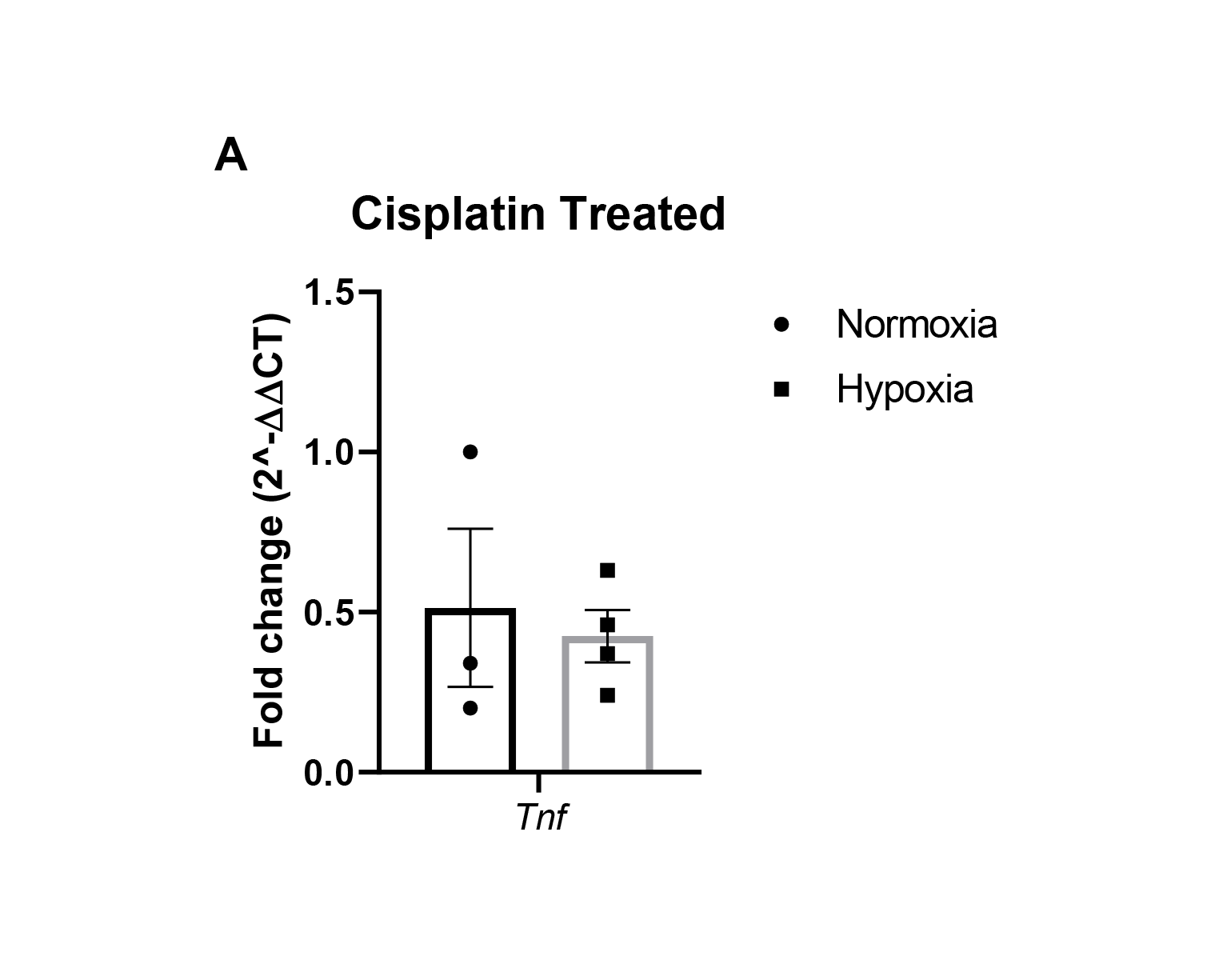

Supplement: S3 Fig — A) Tnf (P = 0.72) gene expression in 7-week kidneys after cisplatin treatment is unchanged between animals exposed to prenatal normoxia or hypoxia; N = 3v4. (TIF) [file pone.0229618.s004.tif]

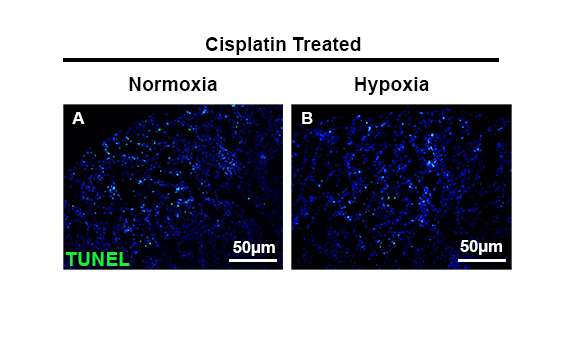

Supplement: S4 Fig — A-B) TUNEL staining on kidneys after cisplatin treatment shows that there are not differences in the number of apoptotic nuclei after injury. (TIF) [file pone.0229618.s005.tif]
